# Dezemberfieber senken: Vermeidung von verschwenderischen Jahresendausgaben

**DOI:** 10.1007/s10273-022-3213-5

**Published:** 2022-06-24

**Authors:** Christoph Siemroth

**Affiliations:** Department of Economics, University of Essex in Colchester, 3.204, Colchester Campus, CO4 3SQ Colchester, UK

**Keywords:** H83, G31

## Abstract

Im öffentlichen Sektor laufen die Finanzmittel zum Jahresende aus, sodass die Verantwortlichen einen starken Anreiz haben, die verbleibenden Mittel kurz vor Ablauf der Frist ineffizient auszugeben. Die Arten von Ineffizienzen dieses Verhaltens, seine Ursachen und dessen Ausmaß werden erörtert sowie eine Lösung vorgeschlagen: Es sollte möglich sein, ungenutzte Mittel teilweise auf das nächste Jahr zu übertragen, wodurch Anreize geschaffen werden, am Jahresende nicht alles zu verschwenden. Der Teil, der nicht übertragen wird, kann zur Unterstützung unterfinanzierter Bereiche verwendet werden.

## Abstract (en)

This article reviews the economic literature on wasteful year-end spending. In the German public sector, budgets expire at the end of the year, hence procurement officers have strong incentives to spend the remaining funds inefficiently just before they expire. The article discusses the kinds of inefficiencies of this behavior, its causes, what is known about its extent, and proposes a solution: It should be possible to partially roll-over unused funds to the next year, which provides incentives not to wastefully spend everything at year-end. The part that is not rolled over can be used to support underfunded divisions. For comparison, the existing German public budget rules are briefly discussed.

Das Haushaltsrecht der deutschen öffentlichen Finanzen basiert bis auf Ausnahmen auf den Prinzipien der „Jährlichkeit“ und „zeitlicher Bindung“: Die Finanzmittel stehen für ein Jahr zur Verfügung und verfallen, wenn sie ungenutzt bleiben. Es gibt Anekdoten, die von Beamt:innen erzählen, die im Dezember noch panisch versuchen, ungenutzte Mittel auszugeben. Eine Sorge ist, dass diese Ausgaben entweder unnötig sind oder wegen des Zeitdrucks schlecht ausgeführt werden, etwa weil die besten Anbietenden so kurzfristig nicht liefern können. Mit anderen Worten, ein Teil der öffentlichen Mittel wird verschwendet. Das Phänomen besteht weltweit und ist so bekannt, dass es hierzulande seinen eigenen Namen hat, „Dezemberfieber“. In Kanada endet das Fiskaljahr im März, dort wurden die exzessiven Jahresendausgaben als „March Madness“ bezeichnet. In den USA heißt das Phänomen „use it or lose it“. Dort campieren Vertriebsleute förmlich in der letzten Woche des Fiskaljahres in Ministerien, falls doch noch schnell ein paar Millionen auszugeben sind (Liebman und Mahoney, 2017).

## Arten und Probleme der Verschwendung

In den USA kommt es regelmäßig zu einem Aufschrei, wenn öffentliche Finanzmittel vermeintlich verschwendet wurden, etwa wenn das Pentagon kurz vor Jahresende Millionen für Hummer ausgibt oder einen Bürostuhl für 9.000 US-$ kauft (Military Times, 2019). Eine Art von Verschwendung sind somit Ausgaben, die nicht oder günstiger getätigt worden wären, wenn die Finanzmittel nicht verfallen würden. Eine weitere Art besteht darin, dass zwar nicht mehr ausgegeben wird, aber wegen des Zeitdrucks am Jahresende eine geringere Qualität gekauft wird. In Deutschland waren Trainingsprogramme für Arbeitslose, die nur wegen verbleibender Mittel am Ende des Jahres durchgeführt wurden, wirkungslos (Fitzenberger et al., 2016), obwohl die Programme normalerweise durchaus die Arbeitslosigkeit senken. Liebman und Mahoney (2017) zeigen für die USA, dass die Qualität von IT-Projekten, die in der letzten Woche des Jahres geordert wurden, später zwei bis sechs Mal häufiger schlecht bewertet wurde als die von Projekten, die vorher geordert wurden.

Beschaffungsbeamte versuchen teils, die Regeln in bester Absicht zu umgehen. Eine Buchprüfung in Missouri bemängelte, dass am Jahresende Vorauszahlungen für Güter und Dienstleistungen geleistet wurden, die erst Monate oder Jahre später erbracht werden sollten (Schweich, 2012). Die Verschwendung besteht hier in dem Risiko, dass die Leistung nicht erbracht wird, weil die Dienstleister vorher bankrott gehen. Oder die Mittel werden für haltbare Güter ausgegeben, die hoffentlich später noch einen Nutzen haben. Einem kanadischen Offizier wurde z.B. befohlen, eine Zugladung Klopapier zu kaufen, bevor die Mittel verfallen (Hurley et al., 2014). Diese Anschaffung ist nicht komplett verschwendet, vermutlich kann die Wagenladung später ihrem Zweck zugeführt werden. Aber der Umtausch von Finanzmitteln in Güter ist mit einem Verlust von Wert und Flexibilität verbunden.

In den meisten Fällen würden die öffentlichen Mittel besser verwendet, wenn es entweder die Möglichkeit gäbe, sie

* Ich danke Hans Peter Grüner und Felix Jarman für informative Gespräche zum Thema.

**Dr. Christoph Siemroth** ist Associate Professor an der University of Essex in Colchester, UK.

zeitlich flexibler zu nutzen, also in der gleichen Abteilung zu einem späteren Zeitpunkt, wenn mehr Anschaffungen nötig sind. Oder wenn es die Möglichkeit gäbe, ungenutzte Mittel zu Abteilungen zu transferieren, die unterfinanziert sind. Könnte man die Jahresendverschwendung verringern, dann könnte der öffentliche Sektor mit den gleichen Mitteln mehr oder könnte das Gleiche mit weniger Mitteln erbringen.

## Ursachen

Unter den existierenden Regeln gibt es kaum Anreize für Abteilungen in Ämtern und Ministerien, nicht alles auszugeben. Tatsächlich gibt es starke Anreize, alles auszugeben, egal, ob das nötig ist, um den Auftrag der Abteilung zu erfüllen oder nicht. Der am häufigsten genannte Grund, warum verschwenderisch alles ausgegeben wird, ist die Befürchtung, dass sonst in den folgenden Jahren die Mittel gekürzt werden. Das will keine Abteilung, denn mit Unterfinanzierung zu kämpfen, ist unangenehmer als mehr als nötig auszugeben. Das sind perverse Anreize. Sparsame werden bestraft, Verprassende unter Umständen sogar mit mehr Mitteln belohnt.

Ein weiterer Grund ist, dass keine Alternative wahrgenommen wird: Abteilungen könnten zwar die Mittel nicht ausgeben, aber „das wäre ja Verschwendung". Aus der Perspektive der Abteilung ist die Nichtnutzung der Mittel eine Verschwendung – die Mittel sind ja dann weg – während aus der Steuerzahlerperspektive stattdessen die Nutzung der Mittel eine Verschwendung ist, solange kein sinnvoller Grund zur Ausgabe besteht. Nur, die Mittel sind bei Nichtnutzung nicht weg; sie können anderswo besser verwendet werden oder die öffentliche Verschuldung senken. Aber die bestehenden Haushaltsregeln sind kaum geeignet, einmal bewilligte Mittel in sinnvollere Zwecke umzuleiten. Auch haben die Abteilungen selbst etwas davon, z. B. eine schönere Büroausstattung oder interessante neue Technologie für sich anzuschaffen, sodass Ausgeben der Nichtnutzung vorgezogen wird.

## Eine Lösung

Ökonomische Modelle erlauben es zu untersuchen, welche Regeln verschwenderische Jahresendausgaben minimieren könnten. Das Modell in Siemroth (2022) zeigt, dass ein Übertrag ungenutzter Mittel in das Folgejahr möglich gemacht werden sollte. Das Modell zeigt auch, dass unter bestimmten Bedingungen kein voller, sondern ein anteiliger Übertrag optimal ist. D. h., statt alle ungenutzten Mittel zu übertragen, stehen z. B. 75 % der ungenutzten Mittel im nächsten Jahr zur Verfügung. Somit haben Abteilungen einen Anreiz, nicht alles ineffizient auszugeben, denn sie können einen Teil der Mittel später verwenden. Der zurückgegebene Teil kann unterfinanzierten Abteilungen helfen. Dieser Vorschlag mag auf Skepsis treffen: Der Übertrag ungenutzter Mittel könnte ja zu einem Überschuss an Geld führen, das ungenutzt bleibt. Aber im schlimmsten Fall werden die Mittel dann später statt früher verschwendet, was nicht schlimmer ist als der Status quo. Im besten Fall werden die Mittel gar nicht mehr verschwendet, sondern nur später sinnvoll genutzt. Es ist also eine Verbesserung, denn nötige Ausgaben können weiterhin sofort getätigt werden, und die zusätzliche Flexibilität vermindert Verschwendung.

Das Modell von Malenko (2019) untersucht frei von institutionellen Beschränkungen, wie eine optimale Kapitalzuweisung an eine Abteilung aussehen würde. Das Ziel dieser Budgetregelung ist, dass die Abteilung sowohl ihren Auftrag erfüllen kann, aber gleichzeitig so wenige Mittel wie möglich verschwendet. Die optimalen Regeln sind wie folgt: Die Abteilung bekommt kontinuierlich Mittel, etwa jeden Monat, die flexibel für Zwecke bis zu einer bestimmten Höhe ausgegeben werden können. Die Mittel verfallen nie, somit gibt es keine verschwenderischen Jahresendausgaben. Man kann das als unbegrenzten und vollen Mittelübertrag interpretieren. Ausgaben ab einer bestimmten Höhe dürfen nicht durch die Abteilung selbst getätigt werden. Sie müssen der Finanzabteilung vorgeschlagen werden, die diese nach einer Prüfung entweder bewilligt und selbst bezahlt oder aber ablehnt und die Abteilung bestraft. Die Bestrafung stellt sicher, dass nur Ausgaben vorgeschlagen werden, die im öffentlichen Interesse sind und wahrscheinlich bewilligt werden, um den Prüfungsaufwand zu senken.[1] Das Bemerkenswerte an dieser Studie ist, dass nichts von „Jährlichkeit" oder „zeitlicher Bindung" in den optimalen Budgetregeln zu finden ist, diese aber in öffentlichen Sektoren vieler Länder verbreitet sind. Die Kosten der dadurch herbeigeführten Inflexibilität und Verschwendung sind schlicht zu hoch, um optimal zu sein.

Die ökonomische Theorie sagt also recht klar, dass Mittelübertrag möglich sein sollte. Und in der Praxis? Ein Pilotprojekt im Justizministerium der USA erlaubte es, ungenutzte Mittel für die IT in Folgejahre zu übertragen und später zu nutzen. Liebman und Mahoney (2017) zeigen, dass diese Maßnahme die Jahresendausgaben für IT um etwa 9 Prozentpunkte senkt, im Vergleich zu anderen Bereichen, bei denen ein Übertrag nicht möglich war. 9 Prozentpunkte klingen nicht viel, aber in anderen Ministerien werden für die IT in der letzten Woche etwa 12 % ausgegeben. Die Reduktion um 9 Prozentpunkte auf 3 % beseitigt die erhöhten Jahresendausgaben damit fast komplett; bei Gleichverteilung würden auf jede Woche 1,9 % entfallen. Auch aus empirischer Sicht ist ein Mittelübertrag also eine vielversprechende Möglichkeit. Dabei ist aber wichtig, dass bei Einsparungen nicht zukünftige Mittel gekürzt werden, denn das würde den Sparanreiz neutralisieren. Das muss glaubhaft kommuniziert werden, sonst wird trotzdem

1 Die Bestrafung könnte etwa eine gestrichene Bonuszahlung sein.

verschwendet. Eine „Steuer“ auf den Übertrag, etwa wenn nur 75 % übertragen werden, kompensieren für zu hohe Budgets, aber nur dann, wenn sie wirklich zu hoch sind.

## Ein einfaches Beispiel

Nehmen wir an, es gibt eine IT-Abteilung einer öffentlichen Einrichtung, die 1 Mio. Euro Mittel im Jahr zur Verfügung hat. Der Auftrag besteht darin, die IT im Haus betriebsfähig zu halten. Das bedeutet etwa Austausch defekter Hardware oder Anschaffung neuer Software bei Bedarf. Am Anfang des Jahres ist es schwer zu sagen, wie viele Computer über das Jahr ersetzt werden müssen. Wenn wenige ersetzt werden müssen, fallen vielleicht nur 0,5 Mio. Euro Ausgaben an, wenn viele ausfallen, reichen die Mittel nicht. Alles dazwischen ist möglich. Die nötigen Ausgaben über das folgende Jahr sind also variabel. Aber die bewilligten Mittel sind unabhängig davon fixiert. Es ist also offensichtlich, dass diese Abteilung, wenn sie jedes Jahr ihre Mittel voll ausschöpft, zumindest in einigen Jahren verschwenderisch ist. Dabei definieren wir jegliche Ausgaben, die nicht nötig sind, als verschwenderisch. Der Mittelübertrag würde helfen, die Ausgaben an den tatsächlichen Bedarf anzupassen. Wenn also nur 0,5 Mio. Euro an Hardware ersetzt werden müssen, gibt es im Dezember die Wahl, die restlichen Mittel irgendwie auszugeben oder nichts auszugeben, aber 75 % der Ersparnis im nächsten Jahr zusätzlich zu bekommen. Das wären insgesamt 1,375 Mio. Euro. Falls dann viel Hardware ersetzt werden müsste, hätte die Abteilung direkt die Mittel, diese zu ersetzen, ohne Anträge auf Extramittel stellen zu müssen. Das ist ein Grund zu sparen, den es andernfalls nicht gibt. Und es wird langfristig weniger Anträge auf Extramittel geben.

## Existierende Regelungen in Deutschland

Tatsächlich sieht die Bundeshaushaltsordnung, ähnlich die Haushaltsordnungen der Länder, in § 19 vor, „Ausgaben können im Haushaltsplan für übertragbar erklärt werden, wenn dies ihre wirtschaftliche und sparsame Verwendung fördert.“ Das bedeutet allerdings nicht, dass die für übertragbar erklärten Mittel dann auch automatisch im nächsten Jahr zur Verfügung stehen. Um ungenutzte Mittel im Folgejahr nutzen zu können, ist der grobe Prozess wie folgt: Ungenutzte Mittel werden für den Übertrag vermerkt, die Beauftragte für den Haushalt (BfdH) muss für das nächste Jahr einen Ausgaberest bilden, anderswo muss der gleiche Betrag im nächsten Jahr eingespart werden, und schließlich muss das Finanzministerium der Nutzung explizit zustimmen. Es gibt also mindestens zwei Vetopunkte: BfdH und Finanzministerium. Außerdem ist im Dezember unklar, ob es im nächsten Jahr genug Einsparung gibt, um die Nutzung des Übertrags zu finanzieren, und das, obwohl der Betrag ja im Vorjahr schon gespart wurde. Böse Zungen mögen nun sagen, eine Abteilung hat bessere Aussichten, nächstes Jahr mehr Mittel zu bekommen, wenn sie Lottoscheine kauft, statt Mittel zu sparen. Zumindest ist der Prozess unnötig umständlich. Mit dieser Unsicherheit und den Hürden ist es für Abteilungen recht unattraktiv, Mittel zu sparen und zu übertragen. Es ist einfacher, die Mittel am Jahresende auszugeben. So führt diese Regelung wohl zu mehr Verschwendung, als wenn ungenutzte Mittel unkompliziert und sicher übertragen werden können.

Die gute Nachricht ist, dass das Bundeshaushaltsgesetz die Übertragregeln in den vergangenen Jahren in bestimmten Fällen flexibler gemacht hat. Es wurden „flexibilisierte Töpfe“ eingeführt und ausgeweitet. Ungenutzte Mittel sind in diesen übertragbar. Ein Einverständnis vom Finanzministerium wird seit 2016 pro forma gegeben (BMF, 2015). Und das Ressort muss nicht selbst im nächsten Jahr sparen, um gesparte und übertragene Mittel nutzen zu können. Die Anreize, in flexibilisierten Töpfen zu sparen, sind stärker. Seit 2021 gilt zudem der vorgeschlagene anteilige Übertrag, sodass in flexibilisierten Töpfen im nächsten Jahr maximal 85 % der gesparten Mittel genutzt werden können (ZFB, 2021, 3.2). Leider gibt es weniger entsprechende flexible Regelungen in den Länderhaushaltsgesetzen, der Bund ist zumindest gesetzgeberisch Vorreiter.

Es gibt also teilweise schon gesetzliche Regelungen, die dem Vorschlag in diesem Artikel nahekommen. Aber das gilt hauptsächlich auf Bundesebene in bestimmten Fällen, und ist in der Praxis mit vielen Hürden verbunden. Außerdem ist unklar, ob ein Übertrag in den Unterabteilungen innerhalb der Ressorts möglich ist, wo viele Ausgabenentscheidungen getroffen werden. Wenn nur das Ressort und nicht die einzelnen Abteilungen Mittel zum nächsten Bundeshaushalt übertragen können, dann wird es weiterhin Verschwendung am Jahresende geben. Der Übertrag muss auch auf den untersten Ausgabeebenen unkompliziert möglich sein. Hier sind die Ministerien und Ämter selbst in der Pflicht, die Möglichkeiten der Haushaltsgesetze und -ordnungen auch intern zu nutzen.

## Ausmaß

Das genaue Ausmaß der Verschwendung ist schwer zu bestimmen. Wäre es einfach zu beobachten, dann wäre es auch einfacher zu unterbinden. Insgesamt wird in US-amerikanischen Ministerien für Beschaffung im Vergleich zu einer durchschnittlichen Woche fast fünf Mal so viel in der letzten Woche des Fiskaljahres ausgegeben (Liebman und Mahoney, 2017). Nicht alle zusätzlichen Jahresendausgaben sind zwingend verschwendet, da Abteilungen einige legitime Beschaffungen bewusst zurückhalten können, bis sie wissen, dass keine wichtigeren Ausgaben anfallen. Die Studie zeigt aber auch deutlich, dass ein großer Teil davon verschwendet wird. Die Reduktion von 12 % auf 3 % Ausgaben in der letzten Woche, wenn Übertrag möglich ist, ist die einzige ernsthafte Schätzung der Jahresendverschwendung in der Literatur. In Großbritannien sind Kapitalausgaben durch die Regierung im letzten Monat etwa doppelt so hoch wie sonst in durchschnittlichen Monaten (Baumann, 2019).

Für Deutschland sind nur Quartalszahlen verfügbar, die den Jahresendeffekt aber etwas verwaschen. Der Vergleich der Ausgaben für den laufenden Sachaufwand vom letzten Quartal und der ersten drei Quartale ist Tabelle 1 zu entnehmen. Diese Ausgaben enthalten Verwaltungsausgaben ohne Personal,[2] wie etwa die Beschaffung von Geräten und Ausrüstung, von denen wir erwarten, dass sie besonders von der Jahresendverschwendung betroffen sind. Die Tabelle weist den Quotienten aus den Ausgaben im letzten Quartal und dem Durchschnitt der Ausgaben in den vorherigen Quartalen separat für Bund, Länder und Gemeinden aus. Im Durchschnitt über fünf Jahre geben der Bund 72 % mehr im letzten Quartal aus, die Länder 34 % und die Gemeinden 13 %. Für alle Ebenen und alle Jahre wird im letzten Quartal deutlich mehr ausgegeben als in denen davor. Die Unterschiede zwischen Bund und Gemeinden sind besonders groß. Wäre der Quotient durchgängig 1, dann wäre es stark evident, dass verschwendete Jahresendausgaben kein Problem sind. So aber kann nicht ausgeschlossen werden, dass diese Verwendung sehr verbreitet ist.

Tabelle 1
**Ausgaben im 4. Quartal im Verhältnis zum Durchschnitt der Ausgaben der ersten drei Quartale**

| | Bund | Länder | Gemeinden |
|---|---|---|---|
| 2019 | 1,776 | 1,283 | 1,086 |
| 2018 | 1,823 | 1,293 | 1,106 |
| 2017 | 1,694 | 1,446 | 1,088 |
| 2016 | 1,622 | 1,258 | 1,182 |
| 2015 | 1,698 | 1,411 | 1,170 |
| Durchschnitt | 1,723 | 1,338 | 1,127 |

Quelle: eigene Berechnungen mit Daten der statistischen Ämter des Bundes und der Länder.

## Fazit

Öffentliche Haushalte stehen unter Druck. Die demografische Entwicklung strapaziert die Sozialkassen. Der Verteidigungshaushalt wurde zuletzt massiv erhöht. Vor diesem Hintergrund ist es wichtig, dass öffentliche Finanzmittel nicht verschwendet werden. Ungenutzte Mittel sollten anteilig ins nächste Jahr übertragen werden können, denn so gibt es einen Anreiz, nicht alles zu verschwenden, wenn Mittel übrig sind.

Die Öffentlichkeit merkt, wenn bestimmte öffentliche Dienste unterfinanziert sind und nicht funktionieren. Aber nur eine detaillierte Rechnungsprüfung zeigt, ob funktionierende öffentliche Dienste auch günstiger hätten erbracht werden können. Es braucht also Regeln, die effizientes Ausgabenverhalten begünstigen. Der unkomplizierte, anteilige Übertrag ungenutzter Mittel ist eine solche Regel. Formal ist das in Ausnahmen in Bundes- und Länderhaushalten möglich, aber es sind viele Hindernisse vorhanden und die Regelungen erstrecken sich nicht automatisch über alle Abteilungen der Ministerien und Ämter. Es gibt Verbesserungspotenzial.

2 Somit sind die Mehrausgaben im letzten Quartal nicht durch „Weihnachtsgeld" zu erklären. Der Betrachtungszeitraum umfasst die vergangenen fünf Jahre, bevor die COVID-19-Pandemie ab Mitte 2020 außergewöhnliche Ausgaben verursachte.
